# Design of a Bacteriophage‐Based Product for Biocontrol of Fire Blight and Black Rot

**DOI:** 10.1111/1751-7915.70436

**Published:** 2026-09-02

**Authors:** Gloria Vique, Pedro Blanco‐Picazo, Aina Trenchs, Maria Dolores Ramos‐Barbero, Maria Isern, Pablo Quirós, Sergio Atares, Ignasi Salaet, Laura Sala‐Comorera, Lorena Rodríguez‐Rubio, Maite Muniesa

**Affiliations:** ^1^ Departament de Genètica, Microbiologia i Estadística Universitat de Barcelona Barcelona Spain; ^2^ Departamento de I+D+i de Fertinagro Biotech S.L Polígono Industrial La Paz Teruel Spain; ^3^ Institut d'Investigació en Nutrició i Seguretat Alimentària (INSA) Universitat de Barcelona Santa Coloma de Gramenet Spain

**Keywords:** bacteriophages, biocontrol, *Erwinia*, lysis, persistence, photoprotectant, *Xanthomonas*

## Abstract

Controlling phytopathogenic bacteria is essential for safeguarding crops and minimizing economic losses in agriculture. Although chemical pesticides are effective, they have adverse effects on ecosystems, beneficial organisms such as pollinators, and natural pathogen predators, creating a need for sustainable alternatives. Bacteriophages ɸEF1 and ɸEF2 (infecting *Erwinia amylovora* , the fire blight pathogen), and ɸXF1 (targeting *Xanthomonas campestris* , the black rot pathogen) have shown promise as biocontrol agents in vitro and in vivo. However, to be effective in the field these phages must maintain infectivity during storage and environmental exposure. We evaluated their stability under varying pH, temperature, UV and sunlight conditions and assessed various photoprotective additives. The phages remained infectious for up to 3 months at 4°C and 20°C and pH 7–9, but were inactivated at pH 3 and 37°C. UV rapidly inactivated the phages, but their stability was significantly enhanced by adding photoprotectants like Amino 22% or CaCO_3_, particularly CaCO_3_ at 24%, which preserved infectivity for over 8 h. These photoprotectants also improved phage recovery from plant surfaces. We conclude that effective biocontrol formulations should contain ≥ 10^6^ pfu/mL, maintain neutral pH, include 24% CaCO_3_, and allow room temperature storage. This study is among the first to demonstrate the long‐term stability of phage‐based formulations at room temperature, together with improved field persistence, supporting their potential use as biopesticides.

## Graphical abstract

Phytopathogenic bacteria threaten crops, and while chemical pesticides are effective, they harm ecosystems. Bacteriophages like ɸEF1, ɸEF2 and ɸXF1 show promise as biocontrol agents. They remain stable at 4°C and 20°C under neutral conditions, with stability enhanced by additives like CaCO_3_, improving field effectiveness for ecological and sustainable crop protection. Some graphical elements have been adapted from Freepik (freepik.com).

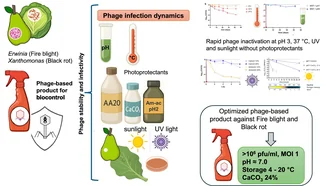

## 1 Introduction

Bacterial phytopathogens represent a major challenge to modern agriculture, causing substantial economic losses and contributing to environmental degradation (Martins et al. 2018). Although chemical pesticides remain widely used because they are effective and easy to manufacture, apply and store (Haq et al. 2024), their application has considerable drawbacks. These include soil contamination, reduced soil fertility and the leaching of toxic compounds into groundwater and aquifers. Some chemical sprays and seed treatments contain copper compounds that accumulate in the environment, and the emergence of copper resistance in target bacteria has reduced their effectiveness (Qiao et al. 2021). Additionally, the non‐specific mode of action of many pesticides disrupts beneficial microbial communities and eliminates natural predators of plant pathogens (Pathak et al. 2022), sometimes promoting pathogen proliferation after treatment.

Chemical pesticides can also endanger pollinators, including honeybees, through direct exposure, residue accumulation and habitat contamination. Given the crucial role pollinators play in the yield and quality of fruit, nut and oilseed crops, their decline poses a direct risk to agricultural productivity (Janousek et al. 2023). Antibiotics have also been used to control phytopathogens, but the global rise of antibiotic resistance and the associated risks for human health limit their suitability for widespread agricultural use (Batuman et al. 2024). Moreover, resistance development in phytopathogens reduces the long‐term efficacy of antibiotic treatments.

Consequently, most conventional pesticides and antibiotics are being banned by the European Union (Gensch et al. 2024), and new control tools are needed for farmers. In response to these limitations, alternative strategies to combat phytopathogens have been developed in recent years (Haq et al. 2024), including the use of biological control agents such as beneficial bacteria, fungi and bacteriophages. Biocontrol agents offer a safer and more sustainable alternative to chemical treatments, as they leave no harmful residues in the environment and pose a lower risk to non‐target organisms.

Among these approaches, phage‐based products constitute a particularly promising and innovative strategy for controlling bacterial plant diseases (Choudhary et al. 2025). Phages are viruses that infect and lyse their specific bacterial hosts, without affecting beneficial microorganisms, plants, animals or humans. Due to this host specificity, phage applications are not expected to adversely alter plant or soil microbiota. Moreover, phages replicate only in the presence of sufficient numbers of susceptible bacteria and naturally decline once the phytopathogenic populations decrease.

In a previous study, two virulent phages targeting *Erwinia amylovora* and one phage infecting *Xanthomonas campestris* pv. *campestris* were isolated and characterized (Vique et al. 2025). These phages showed effectiveness against their respective hosts both in vitro and in vivo under laboratory conditions, using pear and kohlrabi infection models for *E. amylovora* and *X. campestris* , respectively. Species of *Xanthomonas* and *Erwinia* affect a wide range of economically important crops, including cereals, citrus fruits, cruciferous vegetables, tomatoes, beans and other fruits (Timilsina et al. 2020). In particular, *X. campestris* and *E. amylovora* rank among the top ten most significant phytopathogens worldwide (Mansfield et al. 2012). *X. campestris* causes black rot, a disease that primarily affects cruciferous crops such as cabbage, broccoli and cauliflower (Vicente and Holub 2013), while *E. amylovora* is the causal agent of fire blight, which impacts several plant species in the *Rosaceae* family, including fruit trees and ornamental plants (Piqué et al. 2015). Under the current EU plant health regulation 2024/3115 (https://eur‐lex.europa.eu/eli/reg/2024/3115/oj), fire blight ( *E. amylovora* ) is classified as a regulated non‐quarantine pest in most member states and is a quarantine pest status in designated protected zones. In the absence of effective curative treatments, official control measures typically require the prompt removal and destruction of infected plants or plant parts upon detection of symptoms.

The application of phages as biocontrol agents has the potential to reduce or even replace chemical treatments, thereby contributing to a more sustainable agriculture. However, phage performance under controlled laboratory conditions, where phage‐host interactions are typically assessed in liquid cultures, does not necessarily translate to efficacy in greenhouse or field settings. The development of a practical phage‐based product therefore requires evaluation of parameters that influence infection efficiency, environmental stability, persistence on plant surfaces, storage viability and formulation design. In this study, we systematically assessed these factors to optimize a phage formulation for the control of bacterial phytopathogens.

## 2 Experimental Procedures

### 2.1 Bacterial Strains, Bacteriophages and Culture Conditions

The bacterial strains *E. amylovora* NCPPB 595 CECT 222 and *X. campestris* pv. *campestris* CECT 97 were used as hosts for the propagation of bacteriophages. Cultures were grown in Nutrient Broth (NB) (Millipore Bedford, MA, USA) at 30°C with shaking.

The phages used in this study were ɸEF1 (GenBank accession no. PP341298) and ɸEF2 (PP356685), both infecting *E. amylovora* NCPPB 595, and phage ɸXF1 (PP475461), infecting *X. campestris* CECT 97.

### 2.2 Phage Enumeration and Infection Dynamics

Phage titers were determined using the double‐layer agar method (Adams 1959) with the corresponding host strains in NB. Briefly, phage suspensions were serially diluted in phage buffer (22 mM KH_2_PO_4_, 50 mM Na_2_HPO_4_, 85 mM NaCl, 1 mM MgSO_4_ and 0.1 mM CaCl_2_). One milliliters of each dilution was gently mixed with 1 mL of the host culture grown in NB until the exponential growth phase (OD_600_ of 0.3) and 2.5 mL of molten NB soft agar (0.7%). The mixture was poured onto NB agar plates (1.5%) and allowed to dry before incubation at 30°C for 18 h. Negative controls included NB alone, phage buffer alone, phage suspension alone and host strain alone.

To optimize infections, phage suspensions were tested against cultures at diverse concentrations of phages (from 10^6^ to 10^9^ pfu/mL) and bacterial cultures (from 10^5^ to 10^9^ cfu/mL) to generate multiplicities of infection (MOI) of 0.01, 0.1, 1 and 10. The number of colony forming units per milliliter (cfu/mL) of each phytopathogen was determined in NB agar. All results are the average of three independent experiments.

For persistence experiments, phages were further propagated in NB cultures using the corresponding host bacteria to generate larger volumes of suspensions for subsequent analysis.

### 2.3 Phage Stability Under Different pH and Temperatures

The persistence of infectious phages ɸEF1, ɸEF2 and ɸXF1 was evaluated using the double‐layer agar method with their respective host strains following exposure to different temperatures, pH values and radiation sources (UV and natural sunlight). Treatments were applied on agar plates and on the surfaces of cabbage leaves ( *Brassica oleracea* ) and pear fruits ( *Pyrus communis* cv. Ercolini). All assays consisted of three independent experiments performed in duplicate.

#### 2.3.1 Effect of Temperature

Phage infectivity after incubation at different temperatures was tested by plaque formation on the corresponding bacterial hosts. Phage lysates (10^8^ pfu/mL) were stored in 10 mL tubes, kept in the dark at temperatures of 4°C, 20°C or 37°C, and sampled at different times for 6 months (Day 0, 1, 3, 7, 14, 21, 28, 42, 60, 120, 150 and 180). Phages in the samples were then enumerated using the double‐layer agar method.

#### 2.3.2 Effect of pH

Phage lysates (10^8^ pfu/mL) were adjusted to pH 3, 7 or 9 using 37% HCl for acidification or 1 N NaOH for alkalization. The samples were incubated in 10 mL tubes at 4°C in the dark and sampled at different time points over 6 months (Day 0, 1, 3, 7, 14, 21, 28, 42, 60, 120, 150 and 180). Before each analysis, the pH of the suspensions was measured using a standard laboratory pH meter (Thermo Scientific, Waltham, MA, USA) calibrated with standard buffer solutions. After confirming the pH, infectious phages were quantified by the double‐layer agar method as described above.

### 2.4 Phage Persistence Under UV Light and Natural Sunlight in the Presence of Photoprotective Agents

Natural sunlight exposure assays were conducted outdoors at different times between sunrise and sunset. Environmental temperature and solar irradiation data were obtained from the regional meteorological service database (Servei Meteorologic de Catalunya, Catalonia, Spain; https://www.meteo.cat/). Phage suspensions (20 mL, 10^6^ pfu/mL) were dispensed in open, sterile 90‐mm Petri dishes and placed in full direct sunlight. Samples were recovered after 0, 2, 4 and 6 h of exposure for enumeration of infectious phages. To eliminate airborne contaminants prior to enumeration, recovered phage suspensions were filtered through low‐protein‐binding 0.22 μm pore‐size membranes (Millex‐GP; Millipore Bedford, MA, USA). Phages in the filtrate were then enumerated using the double‐layer agar method.

### 2.5 Evaluation of Photoprotective Agents

Three biostimulants were assessed for their capacity to protect phages from photoinactivation without interfering with infectivity:

1. Aminic acid, an acidic protein hydrolysate obtained by hydrolyzing animal proteins (blood, hair or feathers) with H_2_SO_4_. The process breaks down complex proteins into amino acids, peptides and ammonium salts, producing a nitrogen‐rich solution. This product is commonly used as an organic fertilizer and plant biostimulant.
2. Amino 22% (Fertinagro Biotech), a commercial levorotatory amino acid‐based fertilizer (22% w/w free amino acids), protects plants against environmental stressors and stimulates photosynthesis (Henderson et al. 2025).
3. Vegepron Sun (UPL), a foliar fertilizer based on calcium carbonate (CaCO_3_), forms a reflective film that lowers the surface temperature of fruits and leaves and prevents sunburn (da Silva et al. 2019).

To select the most effective photoprotectant, preliminary assays under natural sunlight were conducted using ɸXF1 as a model. Phage suspensions were diluted 1:10 in sterile water (control) or photoprotectant solutions to obtain a final concentration of 10^6^ pfu/mL. Amino 22% (AA22) and Vegepron Sun (CaCO_3_) were tested at 24% (v/v), prepared with distilled water. CaCO_3_ shows a neutral pH and could be stored at room temperature. AA22 shows a neutral pH, but each batch was used only until visible precipitation occurred (typically after 2 months of storage), at which point it was replaced by a new batch to avoid precipitates interfering with phage infection. Due to its acidic pH (2.0), aminic acid was tested at 8%, 12% and 24% (v/v) with the pH adjusted to 6.0 using 1 N NaOH to minimize pH‐mediated viral inactivation.

Phage suspensions with or without a photoprotectant were placed in open sterile plates and exposed to natural sunlight for 6 h (09:00–15:00) during summer (June–July 2025). Three independent experiments were performed for each condition and phages were analysed after 6 h of exposure. Based on the rapid inactivation observed with aminic acid (see Section 3), this compound was excluded from further testing.

### 2.6 Phage Persistence Under UV Light With Photoprotectants

Subsequent experiments were conducted using AA22 and CaCO_3_ at 12% and 24% (v/v) with all three phages (ϕEF1, ϕEF2, ϕXF1). Phage suspensions (10^6^ pfu/mL), with or without photoprotectants, were placed in open sterile Petri dishes underneath a germicidal 8‐W UV lamp (model G30T8; 253.7 nm wavelength; Sankyo Denki, Tokyo, Japan) (Allué‐Guardia et al. 2014). The lamp was preheated for 15–30 min before each experiment to ensure stable output. Phage samples were transferred to open, sterile 90‐mm Petri dishes and placed 10 cm below the UV light source. Samples were exposed for 0, 1, 2, 4, 6 or 8 h before enumeration of infectious phages. The UV dose was calculated using the equation *D* = *I* × *T*, where *D* is the dose, *I* is the intensity (0.099 mW/cm^2^), and *T* is exposure time in seconds. This rate was calculated to be 0 (0 h), 356.4 (1 h), 712.8 (2 h), 1425.6 (4 h), 2138.4 (6 h) and 2851.2 (8 h) mJ/cm^2^. After exposure, phages in the dishes were then enumerated using the double‐layer agar method. All assays consisted of three independent experiments performed in duplicate.

### 2.7 Phage Persistence on Plant Surfaces Under Natural Sunlight

The persistence of phages on plant surfaces was assessed on detached pear fruits and cabbage leaves. Pears were washed three times with sterile distilled water and disinfected by immersion in 1.5% sodium hypochlorite for 30 min. Cabbage leaves were washed three times with sterile water and air‐dried. The surface of each fruit or leaf was divided into three marked areas. Each area was inoculated with a 25 μL drop containing one of the following: (1) a phage suspension (10^6^ pfu/mL) with ɸEF1 or ɸEF2 for pears and ϕXF1 for cabbage; (2) the same phage suspensions supplemented with CaCO_3_ (24% v/v) as a protective agent; and (3) phage buffer alone (negative control).

Inoculated samples were placed in a sterile plastic box and transferred outdoors to an area fully exposed to sunlight, as described above. Phages were recovered from the samples after 0, 2, 4 and 6 h. At each time point, the inoculated surface area was excised with a sterile scalpel.

For phage quantification, excised tissue was mixed 1:10 (w/v) with 10% beef extract solution at pH 7.2 (Guzmán et al. 2007) and homogenized using the Stomacher (IUL Instruments GmbH) for 5 min. Homogenates were clarified by centrifugation (at 4000× *g* for 5 min). Infectious phage titers were determined using the double‐layer agar assay. Each experiment included three replicates (three pears or three cabbage leaves per treatment) and was performed independently in triplicate.

### 2.8 Statistical Analyses

Statistical analyses and data plotting were conducted using GraphPad Prism 10 (GraphPad Software). Data were log_10_‐transformed, and the reduction in the number of cells was calculated. Data normality was assessed using the Shapiro–Wilk and D'Agostino & Pearson normality tests. Differences in phage persistence between treatments were evaluated using unpaired *t*‐tests. Statistical significance was defined as *p* ≤ 0.05 or when indicated *p* ≤ 0.1.

The decay rates of infectious phage and genomic DNA were modeled using a first‐order decay model, a one‐phase non‐linear decay model, and a segmental linear model.

The first‐order decay model was defined as follows:

$$C_{t}=C_{0}\;e^{-k_{t}}$$

The one‐phase non‐linear model was defined as follows:

$$C_{t}=\left(C_{0}-\text{Plateau}\right)\;e^{-k_{t}}+\text{Plateau}$$

where *C*_*f*_ and *C*_*0*_ are the concentrations of infectious phages (pfu/mL) at time *t* and time 0, respectively, and *k* is the decay rate constant.

The segmental linear model was defined as follows:

$$C_{t}=C_{\text{f0}}\;e^{-k_{\text{ft}}}+C_{\text{s0}}\;e^{-k_{\mathrm{st}}}$$

where *k*_*f*_ corresponds to the fast decay rate and *k*_*s*_ to the slow decay rate. *C*_*f*0_ and *C*_*s*0_ are the initial concentration in the fast and slow decay period respectively.

The goodness of fit for each model, *r* ^2^ and root mean square error (RMSE) were calculated. The best fitting model was chosen based on the Akaike information criterion. The time required for a 90% reduction in the initial concentration (*T*_90_) was calculated by solving the equation of the best fitting model considering *C*_*t*_ equal to one.

## 3 Results

### 3.1 Optimization of Phage Infectivity

In a previous study, three virulent bacteriophages were isolated from urban wastewater: ɸEF1 and ɸEF2, which target the phytopathogenic bacterium *E. amylovora* CECT 222, and ɸXF1, which targets *X. campestris* CECT 97 (Vique et al. 2025). Genomic analysis confirmed that all three phages follow a lytic cycle, which was consistent with the appearance of transparent plaques and complete host lysis in liquid culture within a single working Day (5–8 h) (Vique et al. 2025).

**FIGURE 1:**
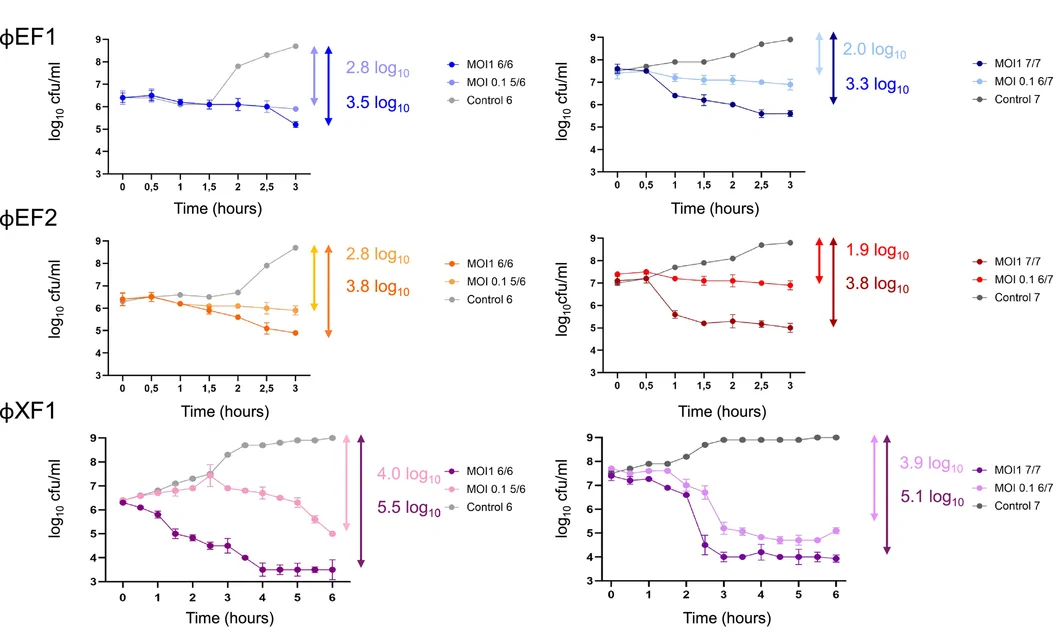
Infectivity dynamics of phages ɸEF1, ɸEF2 and ɸXF1. *E. amylovora* cell counts (cfu/mL) and *X. campestris* pv campestris cell counts (cfu/mL) are monitored along time when infected with the respective phages at MOIs of 0.1 and 1 using initial cell concentrations of 10^6^ and 10^7^ cfu/mL. Control cells correspond to *E. amylovora* and *X. campestris* cultures without the phages. Arrows show logarithmic reductions at the last time of assay.

The infective capacity of each phage was evaluated in vitro at different MOIs. Higher MOIs (10 and 100) did not significantly improve pathogen reduction (Vique et al. 2025). Other studies investigating phages against *E. amylovora* and *X. campestris* have mainly employed MOIs of 0.1 and 1 (Nakayinga et al. 2021; Erdrich et al. 2022; Sabri et al. 2022; Jo et al. 2023; Evseev et al. 2024), although MOIs as high as 10–100 (Biosca et al. 2024) or even 1000 (Choe et al. 2023; Jo et al. 2023) have also been reported. In the present study, a MOI of 1 was selected for subsequent analyses, as it provided greater reductions in host bacterial density compared to an MOI of 0.1 for all three phages (Figure 1). From a practical standpoint, using a lower MOI (1 or 0.1 rather than 100 or 1000) offers significant advantages for product development. It facilitates scalable phage production and allows a single concentrated stock to be diluted for large‐volume formulations. In contrast, higher MOIs would require phage titers of up to 10^10–11^ pfu/mL, substantially increasing manufacturing complexity and cost.

To further evaluate the effect of host density on phage performance, phages were applied at a MOI of 1 to bacterial cultures in the exponential growth phase adjusted to either 10^7^ or 10^6^ cfu/mL. For the three phages, bacterial reduction was equal (ɸEF2) or greater (ɸEF1 and ɸXF1) at the lower host density of 10^6^ cfu/mL compared to 10^7^ cfu/mL (Figure 1). Based on these results, the optimal infection was established with host cultures at 10^6^ cfu/mL and phage suspensions at 10^6^ pfu/mL (MOI = 1).

Under these optimized conditions, phage ɸXF1 exhibited the strongest lytic activity, achieving a 5.5 log_10_ reduction in host density relative to the untreated control within 4 h. This aligns with and confirms prior observations (Vique et al. 2025). Host cell numbers remained stable between 4 and 6 h postinfection, indicating sustained bacterial suppression (Vique et al. 2025). Although their efficacy was lower than that of ɸXF1, phages ɸEF1 and ɸEF2 also caused substantial reductions in host populations, ɸEF1 a 3.5 log_10_ and ɸEF2 3.8 log_10_ reduction when assayed individually. Their synergic use may even increase the host cell reduction, as previously reported (Vique et al. 2025).

### 3.2 Phage Stability at Different pH and Temperatures

**FIGURE 2:**
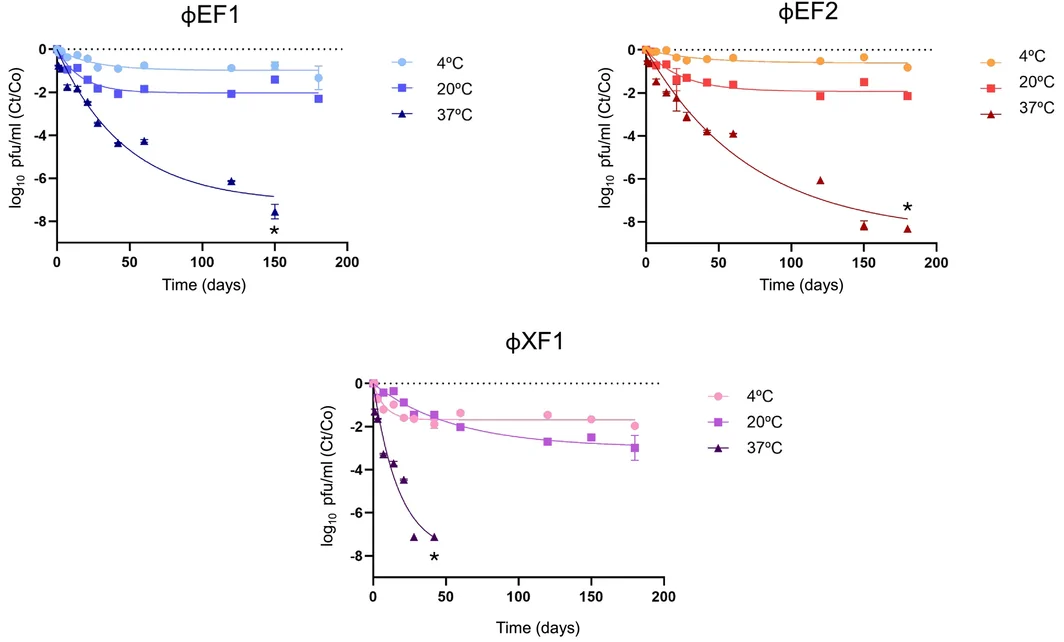
Stability of infectious phages ɸEF1, ɸEF2 and ɸXF1 at different temperatures. Infectious phages (pfu/mL) were evaluated at 4°C, 20°C and 37°C for 180 days of storage. The results are presented as the logarithm of the concentration of the time indicated between the initial concentration. The symbols represent the mean, and the error bars represent the standard deviation. Asterisk indicates results below the detection limit.

Phages ɸEF1 and ɸEF2 remained stable for up to 180 days, with reductions close to or below 1 log_10_ unit at 4°C and 2 log_10_ units at 20°C (Figure 2), these levels are considered acceptable for long‐term storage of phage‐based products. Phage ɸEF1 decay followed a segmental linear regression model at 4°C, with an initial reduction of 0.8 log (initial phase *k* = −0.0266 days^−1^, *T*_90_ = 37.5 days) and then remained stable until the end of experiment (slow phase *k* = −0.0022 days^−1^, *T*_90_ = 460.2 days) (Table S1). At 20°C, ɸEF1 follows a one‐phase decay model (*k* = 0.0609 days^−1^, *T*_90_ = 11.1 days). Phage ɸEF2 remained constant at 4°C with a 0.82‐log_10_ reduction in 6 months and followed a one‐phase decay model at 20°C (*k* = 0.0414 days^−1^, *T*_90_ = 17.5 days) (Table S1). Under incubation at 37°C, both phages showed 8‐log_10_ reduction by day 150 (*T*_90_ = 7.1 and 9 days respectively) and fell below the detection limit thereafter (Figure 2). The differences between the conditions of 4°C, 20°C and 37°C at 150 min are significant for both phages (unpaired *t*‐test: ɸEF1 4°C vs. 37°C: *p* value = < 0.0001; ɸEF2 4°C vs. 37°C: *p* value = < 0.0012), as well as between the conditions of 20°C and 37°C (unpaired *t*‐test ɸEF1 20°C vs. 37°C: *p* value = < 0.0001; ɸEF2 20°C vs. 37°C: *p* value = < 0.0138).

Phage ϕXF1 exhibited comparable stability at 4°C and 20°C (unpaired *t‐*test, *p* value = 0.8520) with reductions close to 2.0 log_10_ (*k* = 0.1249 days^−1^, *T*_90_ = 7.2 days) and 3.0 log_10_ units (*k* = 0.0181 days^−1^, *T*_90_ = 22.7 days), respectively, after 180 days (Figure 2, Table S1). In contrast, the phage titre declined much more rapidly at 37°C (*k* = 0.0562 days^−1^, *T*_90_ = 2.4 days) (Figure 2, Table S1), becoming undetectable by titration within 28 days.

**FIGURE 3:**
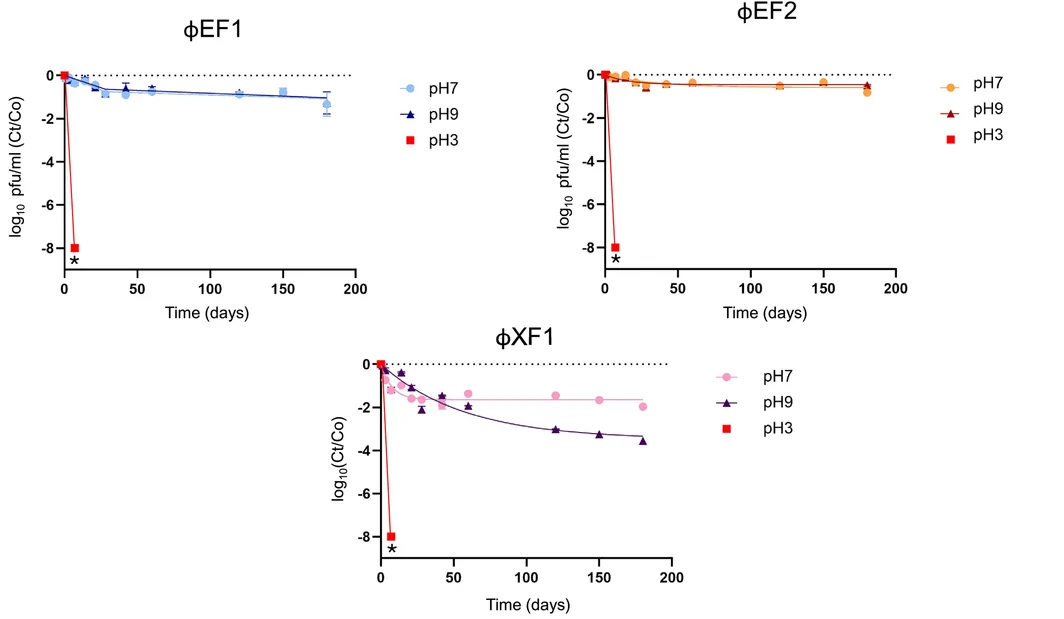
Stability of infectious phages ɸEF1, ɸEF2 and ɸXF1 under different pH. Infectious phages (pfu/mL) were evaluated at pH 3, 7 and 9 during 180 days of storage. The results are presented as the logarithm of the concentration of the time indicated between the initial concentration. The symbols represent the mean, and the error bars represent the standard deviation. Asterisk indicates results below the detection limit.

Acidic conditions also affected the viability of the three phages, which were all inactivated within the first day at pH 3 (Figure 3). At neutral and alkaline pH, stability was significantly higher. Particularly ɸEF2 showed minimal reductions close to 1.0 log_10_ units at both pH 7 and pH 9, without significant differences between these conditions at the different times (unpaired *t*‐test, *p* value = 0.9408). Phage ɸEF1 decreased 1.2 log_10_ units following a segmental linear regression model in both conditions (initial phase *k* = −0.0266 and −0.0226 days^−1^, *T*_90_ = 37.5 to 44.3 days at pH 7 and 9 respectively) (Table S1) and without significant differences between both pHs at different times (unpaired *t*‐test, *p* value = 0.7420). Phage ϕXF1 was less stable decreasing by 2.0 log_10_ units at pH 7 (*T*_90_ = 7.1 days) and 3.8 log_10_ units at pH 9 (*T*_90_ = 19.4 days) (Figure 3 and Table S1) after 180 days of incubation.

### 3.3 Phage Persistence Under UV and Sunlight With Photoprotective Agents

#### 3.3.1 Phage Persistence Under Sunlight With Different Photoprotectants

Environmental factors such as desiccation, temperature and pH, as well as certain chemicals produced by plants can reduce phage viability in the phyllosphere (Buttimer et al. 2017). A primary limiting factor for phage‐based biocontrol is rapid inactivation during daylight, driven by increased temperature and solar UV radiation. UV light induces DNA lesions that can block replication and transcription (Iriarte et al. 2007). To mitigate this effect, UV‐absorbing compounds (e.g., natural extracts from carrot, red pepper and beetroot or amino acids) have been explored as photoprotective agents to prolong phage persistence (Choudhary et al. 2025).

**FIGURE 4:**
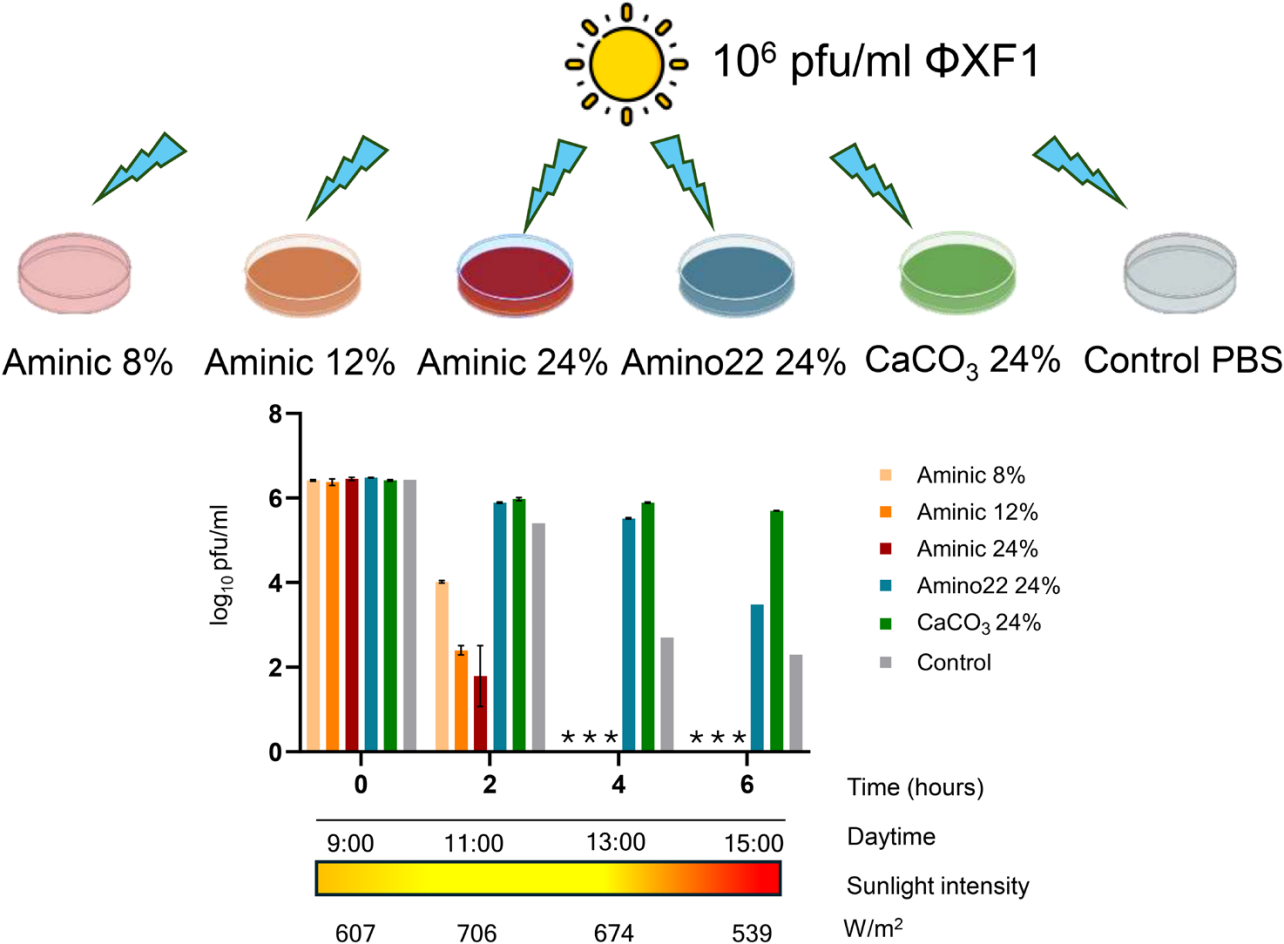
Efficacy of different photoprotectants on phage infectivity. Infectious ɸXF1 phages (log_10_ pfu/mL) were exposed to sunlight for 6 h in the presence of aminic acid (8%, 12% and 24%), AA22 24% and CaCO_3_ 24% as photoprotectants. Control corresponds to the phage suspension without photoprotectant. Sunlight intensity (W/m^2^) was obtained from the Catalan Forecast Service (Servei Meterorològic de Catalunya, Spain) and the average of the intensities along three independent experiments at each given time is indicated. The gradient bar represents daily fluctuations (yellow = highest intensity; red = lower intensity). Error bars represent standard deviation. Asterisk indicates results below the detection limit.

In this study, we evaluated the effect of three photoprotectants (aminic acid, AA22 and CaCO_3_) on phage stability during exposure to natural sunlight for 6 h (9:00 to 15:00). Average sunlight irradiance during this period ranged from 539 W/m^2^ at 15:00 to a peak of 706 w/m^2^ at 11:00 (Figure 4).

Due to its extremely low pH (< 2.0), aminic acid was neutralized with 1 N NaOH to pH 6.0 prior to mixing with the phage suspension to prevent loss of infectious phages due to acidity (as observed in Figure 3). In contrast, AA22 and CaCO_3_ had neutral pH values and did not require buffering.

Even after neutralization, phage suspensions treated with aminic acid declined after 2 h of sunlight exposure and became undetectable by 4 h (Figure 4). This decay exceeded that of the unprotected control suspension, suggesting that residual effects of the initial low pH or the NaOH used for buffering may have contributed to phage inactivation. Supporting this, a more diluted aminic acid solution (8%) showed a smaller reduction in infectious phages after 2 h (2.4 log_10_) than the less diluted solution (24%, 4.1 log_10_ reduction), although infectious phages were no longer detectable beyond this time point. As aminic acid provided little to no phage protection, it was excluded from subsequent experiments.

In contrast, both AA22 and CaCO_3_ provided effective protection against sunlight. The suspension containing 24% CaCO_3_ showed a maximum average reduction of only 0.73 log_10_ units after 6 h of exposure (Figure 4). For AA22‐treated suspensions, reductions were 1.03, 3.73 and 4.13 log_10_ units after 2, 4 and 6 h, respectively (Figure 4). No significant differences were observed between treatments at 2 and 4 h, but the difference at 6 h was significant (unpaired *t*‐test, *p* value = < 0.0001). Based on their protective efficacy, both photoprotectants were included in the next set of experiments.

#### 3.3.2 Phage Persistence Under UV Light

To evaluate photoprotectant efficacy under controlled conditions, suspensions of the three phages were irradiated with a UV lamp in the presence or absence of AA22 and CaCO_3_. To determine an optimal formulation, each agent was tested at two concentrations: 24% as in previous experiments, and a reduced 12% concentration.

**FIGURE 5:**
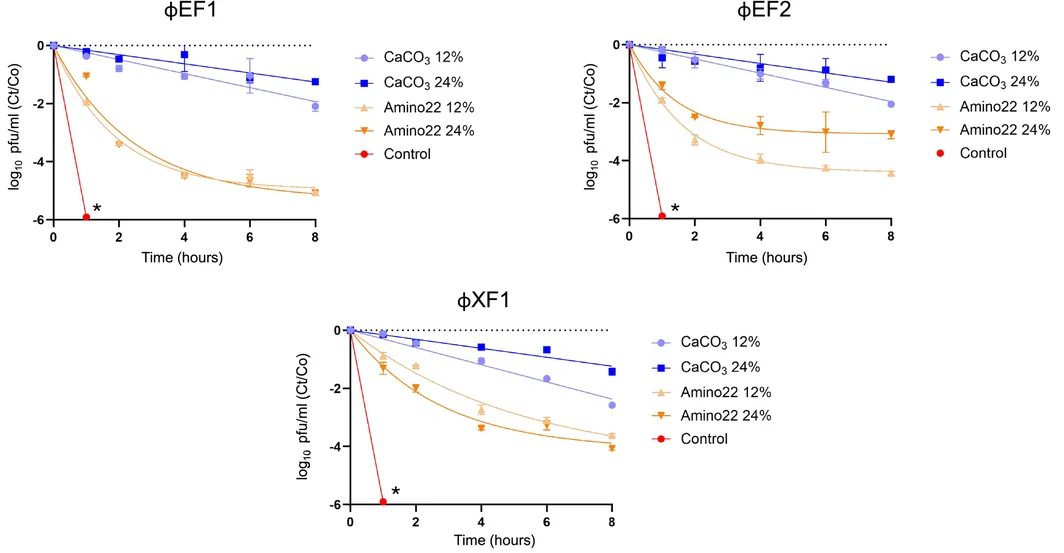
Stability of infectious ɸEF1, ɸEF2 and ɸXF1 phages to UV light. Infectious phages (pfu/mL) were exposed for 8 h to a UV lamp (253.7 nm) using two different photoprotectants (Amino22 and CaCO_3_) at two different concentrations (12% and 24%). Control corresponds to the phage suspensions without photoprotectants. The results are presented as the logarithm of the concentration of the time indicated between the initial concentration. The symbols represent the mean and the error bars represent the standard deviation of three independent experiments. Asterisk indicates results below the detection limit.

Unprotected phage suspensions were rapidly inactivated, falling below the detection limit within 1 h. In contrast, both photoprotectants extended phage viability, with infectious phages recoverable for up to 8 h. Consistent with the sunlight exposure results, CaCO_3_ provided stronger protection than AA22 at different times (Figure 5, Table S2) (unpaired *t*‐test, *p* value = < 0.0001). With CaCO_3_, inactivation was similar for all three phages, with reductions of 1.2–1.4 log_10_ units at 24% and 2–2.5 log_10_ units at 12% at 8 h. Inactivation in the presence of CaCO_3_ followed linear regression models (At 24% *k* = −0.2413, −0.1618 and −0.1547 h^−1^ for phages ɸEF1, ɸEF2 and ɸXF1, respectively).

With AA22, phages ɸEF2 and ɸXF1 were less inactivated than phage ɸEF1 (that reached 5.3 log_10_ units of reduction). Inactivation in the presence of AA22 followed one‐phase decay models (At 24% *k* = 0.4079, 0.7057 and 0.3588 h^−1^ for each phage) (Table S2).

For both agents, the 24% formulation generally provided similar or superior protection compared to the 12% concentration, and the advantage of the higher CaCO_3_ concentration became more pronounced in the last hours of irradiation (Figure 5). The pattern was consistent for all three phages. Given its superior and more consistent mitigation of UV‐induced decay, the 24% CaCO_3_ formulation was selected for incorporation into a phage‐based biocontrol product.

#### 3.3.3 Persistence of Selected Phages on Crop Surfaces Under Sunlight

After the in vitro analyses, we assessed the stability of infectious phages on plant and fruit surfaces exposed to sunlight. This step is essential for translating laboratory efficacy to field applications, as phages that effectively reduce viable cell counts of the host bacteria in vitro often exhibit significant decay on crop surfaces, primarily due to solar radiation (Choudhary et al. 2025). This loss of infectivity can arise from differences in phage adsorption kinetics on plant matrices, which may diminish infection efficiency and overall lytic activity.

To date, most studies on phage interactions with *E. amylovora* have been carried out in standard growth media and buffers, typically using MOIs between 0.01 and 10 (Schnabel and Jones 2001; Gill et al. 2003; Besarab et al. 2020; Kim et al. 2022; Vique et al. 2025). A few recent reports describe successful suppression of *Erwinia* in plants, but many required substantially higher MOIs (10–100) and produced variable outcomes (Sabri et al. 2022; Choe et al. 2023; Biosca et al. 2024).

As noted previously, a key limiting factor for the field application of phage‐based biocontrol is sunlight exposure (Iriarte et al. 2007; Born et al. 2015). Several strategies have been explored to mitigate UV‐induced decay. The simplest is to apply phage preparations in the evening, which extends their persistence in the phyllosphere and increases the likelihood of phages encountering target pathogens before sunrise (Toussaint et al. 2024). Another option is greenhouse application of the phage‐based product, but this limits its utility for outdoor crops. Some approaches aim to select or evolve UV‐tolerant phages (Tom et al. 2018), but this is time‐consuming, yields inconsistent results, and often produces only marginal improvements relative to wild‐type phages. In contrast, the incorporation of photoprotective agents into phage formulations has reliably extended the window of phage infectivity (Born et al. 2015).

**FIGURE 6:**
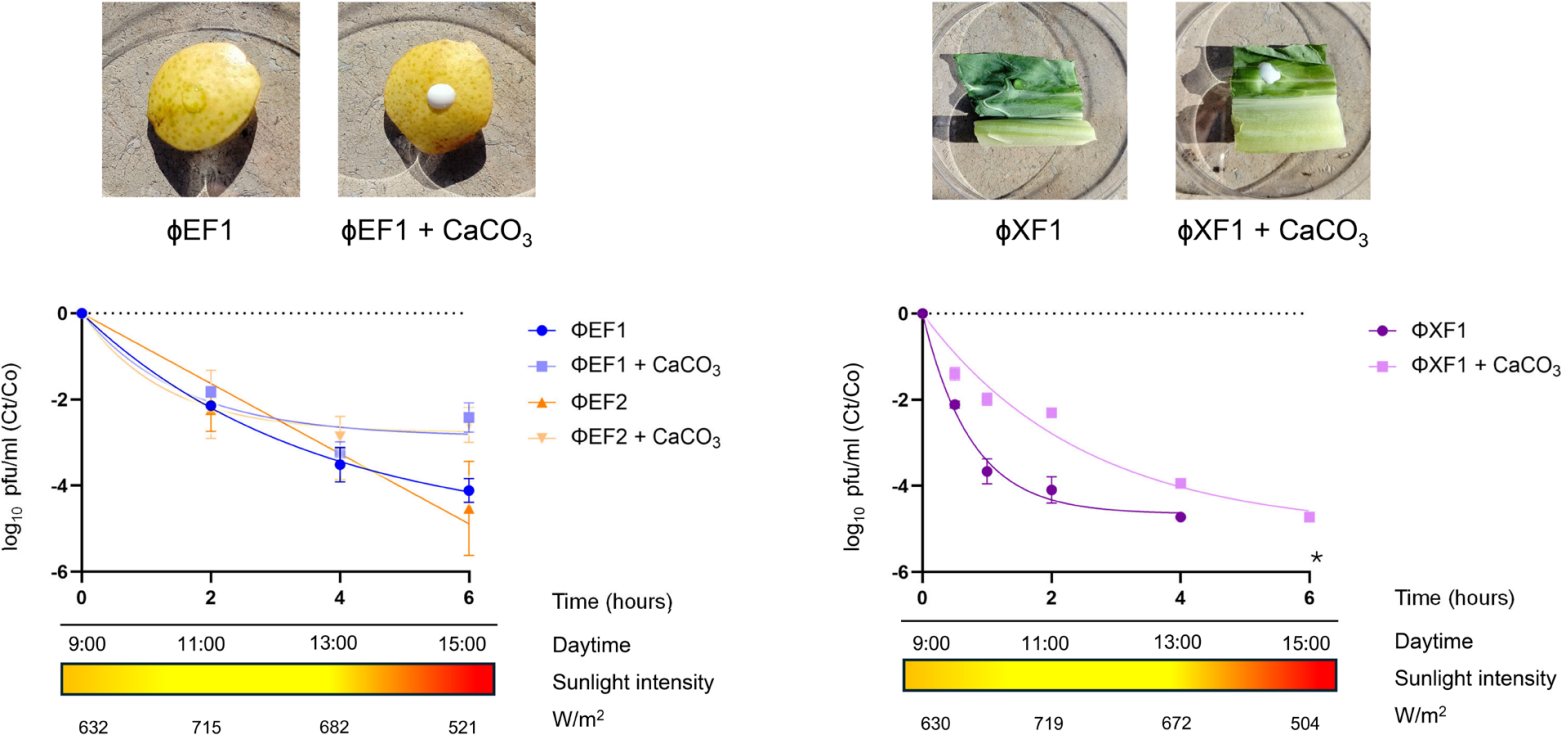
Stability of infectious ɸEF1, ɸEF2 and ɸXF1 phages to sunlight on vegetable surfaces. Infectious phages (pfu/mL) were exposed to sunlight for 6 h in the presence of AA22 24% and CaCO_3_ 24% as photoprotectants. Control corresponds to the phage suspension without photoprotectant. Sunlight intensity (W/m^2^) was obtained from the Catalan Forecast Service (Servei Meterorològic de Catalunya, Spain) and the average of the intensities along three experiments at each given time is indicated. The gradient bar represents daily fluctuations (yellow = highest intensity; red = lower intensity). The results are presented as the logarithm of the concentration of the time indicated between the initial concentration. The symbols represent the mean and the error bars represent the standard deviation of three independent experiments. Asterisk indicates results below the detection limit.

The photoprotectants tested here are already approved for agricultural application. Among the three candidates, CaCO_3_ provided the most promising results, maintaining phage viability on pear slides (for *Erwinia*‐infecting ɸEF1 and ɸEF2) and on cabbage leaves (for *Xanthomonas*‐infecting ɸXF1) (Figure 6). Average sunlight irradiance during the period of pear experiments ranged from 521 W/m^2^ at 15:00 to a peak of 715 w/m^2^ at 11:00 and average irradiances during cabbage experiments from 504 W/m^2^ at 15:00 to a peak of 719 w/m^2^ at 11:00 (Figure 6).

Before evaluating phage persistence, we optimized the protocol for recovering phages from the vegetable surfaces. Among the methods tested, ϕEF1, used as a model for *Erwinia* phages on pear's surface, was evaluated comparing two different homogenization protocols, the homogenization in phage buffer at pH 7 and the second in a beef extract solution 10% at pH 7.2 (Guzmán et al. 2007). Extraction with beef extract yielded the highest recovery, retrieving approximately 76.7% of the theoretical number of phages inoculated on the vegetables, while using phage buffer extraction only 0.79% of the theoretical number of phages was recovered.

The method of homogenization was also evaluated using ϕXF1 and cauliflower leaf. In this case, we compared the use of beef extra and vortex versus beef extra and Stomacher blending. The use of Stomacher blending improved phage recovery by up to 46.47% in comparison to the homogenization by vortex, and consequently, successive experiments were conducted with homogenization using beef extra and the Stomacher device.

As observed in earlier experiments, the addition of 24% CaCO_3_ to the phage suspension improved the recovery of infectious phages from plant surfaces under sunlight after 6 h (unpaired *t*‐test, *p* significance ≤ 0.1 *p* value = 0.0026–0.0902). In pears, after 6 h of exposure, phage ɸEF1 titers were on average 1.8 log_10_ pfu/mL higher in the presence of CaCO_3_ compared to the unprotected control. The protective effect was even more pronounced for phage ɸEF2, which showed a 2.5 log_10_ pfu/mL increase with CaCO_3_.

A similar protective effect was observed for phage ɸXF1 on cabbage leaves. In the presence of CaCO_3_, phage titers increased by 1.8, 1.9 and 1.0 log_10_ pfu/mL at 1, 2 and 4 h post‐application, respectively. In contrast, the unprotected phage suspension fell below the detection limit after 4 h. Attempts to prolong the experiments beyond 6 h were hindered by overnight degradation and desiccation of the plant material, which prevented reproducible measurements. In the presence of CaCO_3_ 24%, all three phages followed the one‐phase decay model (*k* = 0.6391, 0.7788 and 0.4028 h^−1^ for ɸEF1, ɸEF2 and ɸXF1 respectively) (Table S2).

## 4 Discussion

Only a few phage‐based products are commercially available for plant disease management. In Europe, these include Erwiphage PLUS (Enviroinvest, Hungary) (Gayder et al. 2020), which targets *E. amylovora* , and Biolyse (Scotland, https://www.apsbiocontrol.com) for black rot caused by *Xanthomonas* spp. Outside Europe, products such as Ecofire (Ecophage, Israel; https://ecophage.com/), used against *E. amylovora* and fire blight, and AgriPhage (Omnilytics, USA; https://www.omnilytics.com/agriculture/), with formulations for *Xanthomonas* spp. and *E. amylovora* , are in use.

Despite their potential as biocontrol agents, the application of bacteriophages remains limited by several challenges (Holtappels et al. 2021). Their narrow and strain‐specific host range can restrict efficacy against genetically diverse pathogen populations, while the emergence of phage‐resistant bacterial strains may reduce long‐term effectiveness. In addition, phage stability and persistence under field conditions can be affected by environmental factors such as UV radiation, temperature, desiccation and rainfall. Variability in phage–host interactions and formulation performance under different environmental conditions also makes consistent field efficacy difficult to achieve. These limitations highlight the need for improved phage selection, formulation and application strategies to ensure reliable and durable biocontrol performance. While phage‐based products have shown efficacy in specific contexts, and in vitro assays have demonstrated that phages can effectively reduce host population growth for at least 24 h (Nagai et al. 2017; Sabri et al. 2022; Biosca et al. 2024; Vique et al. 2025), their effectiveness in real‐world crop systems can be more variable. Indeed, when applied to crops, some phages may have little impact on pathogen populations or may even result in increased bacterial populations (Grace et al. 2026). These findings highlight the need for additional formulations tailored to pathogen strains prevalent in different geographical and climatic regions. While some broad‐spectrum phages have been isolated, many exhibit a narrow host range. In most environments, phage diversity and host specificity are shaped by local bacterial populations, so that a product developed for one region may show reduced efficacy against strains found elsewhere.

Effective phage‐based biocontrol therefore depends on continued optimization of formulation and deployment strategies. This study advances our understanding of phage‐pathogen dynamics and provides practical insights into product composition and storage conditions to improve phage efficacy in the crops.

An essential step in product development is the evaluation of phage stability and shelf life, as these parameters directly suggest the application strategy. Formulations can be designed for either preventive use, before pathogen colonization or for curative interventions once disease symptoms have already appeared. Given that the current legislation often mandates the removal of infected trees or crops (European Parliament and Council of the European Union 2016), there is a demand for durable preventive formulations.

Storage conditions represent a major challenge for the commercialization of phage‐based products. Temperature is particularly critical, as higher temperatures can reduce both phage viability and infectivity (Kering et al. 2020). For this reason, phages are commonly stored at low temperatures (4°C, −20°C or−80°C), with the longest shelf life achieved under the coldest conditions (Gonzalez‐Menendez et al. 2018). However, dependence on cold storage complicates large‐scale logistics and distribution. Lyophilization (freeze‐drying) offers an alternative (Merabishvili et al. 2013), but the process is costly, requires specialized equipment, and can reduce the number of infectious particles (Choudhary et al. 2025). In this context, this study is among the first to demonstrate long‐term stability of phage formulations at room temperature. This represents a logistical and economical advantage, potentially eliminating the need for cold storage or lyophilization. To further enhance the natural persistence of lytic phages, recent studies have explored the encapsulation of phages in hemicellulose‐based sprays as a strategy to improve their stability during storage, transportation and application, while also potentially enhancing their biocontrol efficacy (Negroni et al. 2026).

Product composition is also of particular concern. In the laboratory, phages are commonly stored in SM buffer, which contains Tris–HCl. However, Tris–HCl is not approved by the FDA as a food additive for novel drugs (Choudhary et al. 2025; USFDA 2025), making it unsuitable for commercial biocontrol applications in agriculture. In contrast, storing phages in culture media, free of hazardous components, may facilitate direct application to crops.

The addition of photoprotective agents to phage formulations can substantially improve outdoor performance by reducing inactivation by sunlight. As the photoprotectants tested in this study (e.g., CaCO_3_) are already approved for agricultural use as biostimulants, their incorporation into phage products is expected to face fewer regulatory barriers. Their inclusion therefore represents a strategic step towards faster authorization and effective deployment of phage‐based biopesticides against plant pathogenic bacteria.

To translate these findings into agricultural practice, specific field application guidelines must be established. Field applications can be performed via spraying, preferentially in the evening (Toussaint et al. 2024) to minimize direct UV exposure during the initial hours of deployment, thereby maximizing the protective window of the CaCO_3_ additive. Given the environmental degradation kinetics observed, a reapplication interval of 5 to 7 days should be recommended during high‐risk periods or after heavy rainfall events. Furthermore, these phage‐based formulations should be designed to maintain compatibility within Integrated Pest Management (IPM) frameworks (Stenberg 2017), ensuring they can be safely co‐applied or rotated with conventional biostimulants and other crop‐protection products without reducing viral infectivity.

From a commercial standpoint, plant protection products based on bacteriophages hold considerable market potential as sustainable alternatives to traditional chemical pesticides (Choudhary et al. 2025). However, their market entry, particularly within the European Union, faces distinct regulatory pathways (Environment directorate, chemicals and biotechnology committee 2022; Czajkowski et al. 2025). Under the current EU regulatory framework, phages are evaluated as microorganisms under the stringent biopesticide approval process (European Parliament and Council of the European Union 2009; Environment directorate, chemicals and biotechnology committee 2022). Navigating these regulatory frameworks requires extensive documentation regarding host specificity and environmental impact. Despite these barriers, the growing demand for residue‐free food and the alignment of phage biocontrol with the objectives of the European Green Deal (European Commission 2019) underscore the substantial commercial viability of these stable, room‐temperature formulations, with enhanced persistence under sunlight, for regional crop protection. Future studies should include greenhouse experiments and multi‐season field trials to validate the efficacy of these phage‐based products under diverse environmental conditions.

## 5 Conclusions

This study demonstrates that the evaluated bacteriophages exhibit robust stability under diverse environmental conditions, supporting their potential as biocontrol agents against phytopathogenic bacteria. Unlike previous studies, our findings provide evidence of the long‐term stability of phage formulations at room temperature and their enhanced persistence on plant surfaces under natural sunlight through the incorporation of CaCO_3_. Collectively, these results demonstrate the feasibility of developing effective phage‐based biopesticides and provide practical guidance for optimizing formulations for field application.

## Author Contributions

**Pablo Quirós:** conceptualization, funding acquisition, writing – review and editing. **Aina Trenchs:** methodology, data curation, writing – review and editing. **Maite Muniesa:** funding acquisition, supervision, project administration, writing – review and editing. **Gloria Vique:** investigation, formal analysis, writing – original draft, writing – review and editing. **Sergio Atares:** conceptualization, funding acquisition, writing – review and editing. **Pedro Blanco‐Picazo:** formal analysis, investigation, writing – review and editing. **Ignasi Salaet:** conceptualization, funding acquisition. **Laura Sala‐Comorera:** formal analysis, methodology, writing – review and editing. **Maria Dolores Ramos‐Barbero:** formal analysis, validation, writing – review and editing. **Maria Isern:** methodology, data curation, writing – review and editing. **Lorena Rodríguez‐Rubio:** supervision, validation, writing – review and editing.

## Funding

This research was funded by the project CPP2023‐010671 funded by MICIU/AEI/10.13039/501100011033 and by ERDF/EU. G.V. has a FPI grant from the Spanish Ministerio de Universidades. M.M. is a researcher of the ICREA Academia 2022 program. M.M. (Serra‐Húnter Professor), L.S.‐C. (Serra‐Húnter Lecturer) and L.R.‐R. (Serra‐Húnter Associate Professor), Generalitat de Catalunya.

## Disclosure

AI usage statement: We hereby confirm that this manuscript was entirely conceived and prepared without the assistance of generative AI tools or large language models. All intellectual content and writing are the original work of the listed authors.

## Ethics Statement

All prevailing local, national, and international regulations and conventions, and normal scientific ethical practices, have been respected.

## Conflicts of Interest

The authors declare no conflicts of interest.

## Supporting information


**Table S1:** Inactivation parameters of phages ɸEF1, ɸEF2 and ɸXF1 at different pHs and temperatures. The best‐fit model (segmental linear regression or one‐phase decay) was selected based on the Akaike information criterion (AIC), *r* ^2^, and root mean square error (RMSE). *k* and *T*_90_ are expressed in days^−1^.
**Table S2:** Inactivation parameters of phages ɸEF1, ɸEF2 and ɸXF1 under UV radiation and natural sunlight (SL) in liquid suspension or on vegetable surfaces (VS) with AA22 or CaCO_3_ at 12% and 24% as photoprotectants. The best‐fit model (Linear regression or one‐phase decay) was selected based on the Akaike information criterion (AIC), *r* ^2^ and root mean square error (RMSE). *k* and *T*_90_ are expressed in hours^−1^.

## Data Availability

The data that support the findings of this study are available from the corresponding author upon reasonable request.

## References

[mbt270436-bib-0001] Adams, M. H. 1959. Bacteriophages. Interscience Publishers.

[mbt270436-bib-0002] Allué‐Guardia, A. , A. Martínez‐Castillo , and M. Muniesa . 2014. “Persistence of Infectious Shiga Toxin‐Encoding Bacteriophages After Disinfection Treatments.” Applied and Environmental Microbiology 80: 2142–2149. 24463973 10.1128/AEM.04006-13 PMC3993145

[mbt270436-bib-0003] Batuman, O. , K. Britt‐Ugartemendia , S. Kunwar , et al. 2024. “The Use and Impact of Antibiotics in Plant Agriculture: A Review.” Phytopathology 114: 885–909. 10.1094/PHYTO-10-23-0357-IA. 38478738

[mbt270436-bib-0004] Besarab, N. V. , A. E. Akhremchuk , M. A. Zlatohurska , et al. 2020. “Isolation and Characterization of Hena1—A Novel *Erwinia amylovora* Bacteriophage.” FEMS Microbiology Letters 367: fnaa070. 32319510 10.1093/femsle/fnaa070

[mbt270436-bib-0005] Biosca, E. G. , R. Delgado Santander , F. Morán , et al. 2024. “First European *Erwinia amylovora* Lytic Bacteriophage Cocktails Effective in the Host: Characterization and Prospects for Fire Blight Biocontrol.” Biology‐Basel 13: 176. 38534446 10.3390/biology13030176 PMC10967764

[mbt270436-bib-0006] Born, Y. , L. Bosshard , B. Duffy , M. J. Loessner , and L. Fieseler . 2015. “Protection of *Erwinia amylovora* Bacteriophage Y2 From UV‐Induced Damage by Natural Compounds.” Bacteriophage 5: e1074330. 26904378 10.1080/21597081.2015.1074330 PMC4743488

[mbt270436-bib-0007] Buttimer, C. , O. McAuliffe , R. P. Ross , C. Hill , J. O'Mahony , and A. Coffey . 2017. “Bacteriophages and Bacterial Plant Diseases.” Frontiers in Microbiology 8: 34. 28163700 10.3389/fmicb.2017.00034 PMC5247434

[mbt270436-bib-0008] Choe, J. , B. Kim , M. K. Park , and E. Roh . 2023. “Biological and Genetic Characterizations of a Novel Lytic Φ106 Against Indigenous *Erwinia amylovora* and Evaluation of the Control of Fire Blight in Apple Plants.” Biology‐Basel 12: 1060. 37626946 10.3390/biology12081060 PMC10452218

[mbt270436-bib-0009] Choudhary, M. , I. A. Bankole , S. T. McDuffee , et al. 2025. “Bacteriophages as Agents for Plant Disease Control: Where Are We After a Century?” Viruses 17: 1033. 40872748 10.3390/v17081033 PMC12390404

[mbt270436-bib-0010] Czajkowski, R. , A. Roca , and M. A. Matilla . 2025. “Harnessing Bacteriophages for Sustainable Crop Protection in the Face of Climate Change.” Microbial Biotechnology 18: e70108. 39937142 10.1111/1751-7915.70108 PMC11816701

[mbt270436-bib-0011] da Silva, P. S. O. , L. F. G. de Oliveira , E. C. de Mattos , et al. 2019. “Calcium Particle Films Promote Artificial Shading and Photoprotection in Leaves of American Grapevines ( *Vitis labrusca* L.).” Scientia Horticulturae 252: 77–84.

[mbt270436-bib-0012] Environment Directorate, Chemicals and Biotechnology Committee . 2022. Guidance Document for the Regulatory Framework for the Microorganism Group: Bacteriophages (Series on Pesticides). Vol. 108. OCDE Publishing.

[mbt270436-bib-0013] Erdrich, S. H. , V. Sharma , U. Schurr , B. Arsova , and J. Frunzke . 2022. “Isolation of Novel *Xanthomonas* Phages Infecting the Plant Pathogens *X. translucens* and *X. campestris* .” Viruses 14: 1449. 35891434 10.3390/v14071449 PMC9316219

[mbt270436-bib-0014] European Commission . 2019. “The European Green Deal.” https://commission.europa.eu/strategy‐and‐policy/priorities‐2019‐2024/european‐green‐deal_en.

[mbt270436-bib-0015] European Parliament and Council of the European Union . 2009. “Regulation (EC) No 1107/2009 of the European Parliament and of the Council of 21 October 2009 Concerning the Placing of Plant Protection Products on the Market and Repealing Council Directives 79/117/EEC and 91/414/EEC.” Official Journal of the European Union. L 309 (24 November): 1–50. Publications Office of the European Union.

[mbt270436-bib-0016] European Parliament and Council of the European Union . 2016. “Regulation (EU) 2016/2031 of the European Parliament of the Council of 26 October 2016 on Protective Measures Against Pests of Plants.” Official Journal of the European Union. L 317 (23 November): 4–104. Publications Office of the European Union.

[mbt270436-bib-0017] Evseev, P. V. , R. I. Tarakanov , H. T. N. Vo , et al. 2024. “Characterisation of New Foxunavirus Phage Murka With the Potential of *Xanthomonas campestris* pv *campestris* Control.” Viruses 16: 198. 38399973 10.3390/v16020198 PMC10892653

[mbt270436-bib-0018] Gayder, S. , M. Parcey , D. Nesbitt , A. J. Castle , and A. M. Svircev . 2020. “Population Dynamics Between *Erwinia amylovora* , *Pantoea agglomerans* and Bacteriophages: Exploiting Synergy and Competition to Improve Phage Cocktail Efficacy.” Microorganisms 8: 1–18. 10.3390/microorganisms8091449 PMC7563384 32971807

[mbt270436-bib-0019] Gensch, L. , K. Jantke , L. Rasche , and U. A. Schneider . 2024. “Pesticide Risk Assessment in European Agriculture: Distribution Patterns, Ban‐Substitution Effects and Regulatory Implications.” Environmental Pollution 348: 123836. 38522603 10.1016/j.envpol.2024.123836

[mbt270436-bib-0020] Gill, J. J. , A. M. Svircev , R. Smith , and A. J. Castle . 2003. “Bacteriophages of *Erwinia amylovora* .” Applied and Environmental Microbiology 69: 2133–2138. 12676693 10.1128/AEM.69.4.2133-2138.2003 PMC154828

[mbt270436-bib-0021] Gonzalez‐Menendez, E. , L. Fernandez , D. Gutierrez , A. Rodríguez , B. Martínez , and P. GarcíaI . 2018. “Comparative Analysis of Different Preservation Techniques for the Storage of *Staphylococcus* Phages Aimed for the Industrial Development of Phage‐Based Antimicrobial Products.” PLoS One 13: e0205728. 30308048 10.1371/journal.pone.0205728 PMC6181408

[mbt270436-bib-0022] Grace, E. R. , V. Milenkovic , K. G. Hinton , et al. 2026. “Characterisation and In Planta Activity of Bacteriophages That Infect the Key Bacterial Species Associated With Acute Oak Decline.” Microbial Biotechnology 19: e70394. 42226654 10.1111/1751-7915.70394 PMC13239085

[mbt270436-bib-0023] Guzmán, C. , J. Jofre , A. R. Blanch , and F. Lucena . 2007. “Development of a Feasible Method to Extract Somatic Coliphages From Sludge, Soil and Treated Biowaste.” Journal of Virological Methods 144: 41–48. 17499367 10.1016/j.jviromet.2007.03.017

[mbt270436-bib-0024] Haq, I. U. , K. Rahim , G. Yahya , B. Ijaz , S. Maryam , and N. P. Paker . 2024. “Eco‐Smart Biocontrol Strategies Utilizing Potent Microbes for Sustainable Management of Phytopathogenic Diseases.” Biotechnology Reports 44: e00859. 39308938 10.1016/j.btre.2024.e00859 PMC11415593

[mbt270436-bib-0025] Henderson, B. C. R. , J. M. Sanderson , and A. Fowles . 2025. “A Review of the Foliar Application of Individual Amino Acids as Biostimulants in Plants.” Discover Agriculture 3, no. 1: 1–14. 10.1007/s44279-025-00222-7 PMC12075294 40376175

[mbt270436-bib-0026] Holtappels, D. , K. Fortuna , R. Lavigne , and J. Wagemans . 2021. “The Future of Phage Biocontrol in Integrated Plant Protection for Sustainable Crop Production.” Current Opinion in Biotechnology 68: 60–71. 33176252 10.1016/j.copbio.2020.08.016

[mbt270436-bib-0027] Iriarte, F. B. , B. Balogh , M. T. Momol , L. M. Smith , M. Wilson , and J. B. Jones . 2007. “Factors Affecting Survival of Bacteriophage on Tomato Leaf Surfaces.” Applied and Environmental Microbiology 73: 1704–1711. 17259361 10.1128/AEM.02118-06 PMC1828813

[mbt270436-bib-0028] Janousek, W. M. , M. R. Douglas , S. Cannings , et al. 2023. “Recent and Future Declines of a Historically Widespread Pollinator Linked to Climate, Land Cover, and Pesticides.” Proceedings of the National Academy of Sciences of the United States of America 120: e2211223120. 36689649 10.1073/pnas.2211223120 PMC9945941

[mbt270436-bib-0029] Jo, S. J. , S. G. Kim , Y. M. Lee , et al. 2023. “Evaluation of the Antimicrobial Potential and Characterization of Novel T7‐Like *Erwinia* Bacteriophages.” Biology‐Basel 12: 180. 36829459 10.3390/biology12020180 PMC9953017

[mbt270436-bib-0030] Kering, K. K. , X. Zhang , R. Nyaruaba , J. Yu , and H. Wei . 2020. “Application of Adaptive Evolution to Improve the Stability of Bacteriophages During Storage.” Viruses 12: 423. 32283683 10.3390/v12040423 PMC7232334

[mbt270436-bib-0031] Kim, S. G. , S. B. Lee , S. J. Jo , et al. 2022. “Phage Cocktail in Combination With Kasugamycin as a Potential Treatment for Fire Blight Caused by *Erwinia amylovora* .” Antibiotics (Basel) 11: 1566. 36358221 10.3390/antibiotics11111566 PMC9686651

[mbt270436-bib-0032] Mansfield, J. , S. Genin , S. Magori , et al. 2012. “Top 10 Plant Pathogenic Bacteria in Molecular Plant Pathology.” Molecular Plant Pathology 13: 614–629. 22672649 10.1111/j.1364-3703.2012.00804.x PMC6638704

[mbt270436-bib-0033] Martins, P. M. M. , M. V. Merfa , M. A. Takita , and A. A. De Souza . 2018. “Persistence in Phytopathogenic Bacteria: Do We Know Enough?” Frontiers in Microbiology 9: 1099. 29887856 10.3389/fmicb.2018.01099 PMC5981161

[mbt270436-bib-0034] Merabishvili, M. , C. Vervaet , J. P. Pirnay , et al. 2013. “Stability of *Staphylococcus aureus* Phage ISP After Freeze‐Drying (Lyophilization).” PLoS One 8: e68797. 23844241 10.1371/journal.pone.0068797 PMC3699554

[mbt270436-bib-0035] Nagai, H. , N. Miyake , S. Kato , D. Maekawa , Y. Inoue , and Y. Takikawa . 2017. “Improved Control of Black Rot of Broccoli Caused by *Xanthomonas campestris* pv. *campestris* Using a Bacteriophage and a Nonpathogenic *Xanthomonas* sp.” Strain. Journal of General Plant Pathology 83: 373–381.

[mbt270436-bib-0036] Nakayinga, R. , A. Makumi , V. Tumuhaise , and W. Tinzaara . 2021. “ *Xanthomonas* Bacteriophages: A Review of Their Biology and Biocontrol Applications in Agriculture.” BMC Microbiology 21: 291. 34696726 10.1186/s12866-021-02351-7 PMC8543423

[mbt270436-bib-0037] Negroni, A. M. , C. G. Hendrich , M. T. Ho , A. Yousefvand , and V. P. Friman . 2026. “Wood Hemicellulose‐Based Spray‐Dried Microencapsulation of a Lytic Bacteriophage Preserves Phage Viability and Improves Control of the Bacterial Wilt Pathogen *Ralstonia solanacearum* .” Microbial Biotechnology 19: e70315. 41688824 10.1111/1751-7915.70315 PMC12904778

[mbt270436-bib-0038] Pathak, V. M. , V. K. Verma , B. S. Rawat , et al. 2022. “Current Status of Pesticide Effects on Environment, Human Health and it's Eco‐Friendly Management as Bioremediation: A Comprehensive Review.” Frontiers in Microbiology 13: 962619. 36060785 10.3389/fmicb.2022.962619 PMC9428564

[mbt270436-bib-0039] Piqué, N. , D. Miñana‐Galbis , S. Merino , and J. M. Tomás . 2015. “Virulence Factors of *Erwinia amylovora* : A Review.” International Journal of Molecular Sciences 166: 12836–12854. 10.3390/ijms160612836 PMC4490474 26057748

[mbt270436-bib-0040] Qiao, K. , Q. Liu , Y. Xia , and S. Zhang . 2021. “Evaluation of a Small‐Molecule Compound, n‐Acetylcysteine, for the Management of Bacterial Spot of Tomato Caused by Copper‐Resistant *Xanthomonas perforans* .” Plant Disease 105: 108–113. 33175655 10.1094/PDIS-05-20-0928-RE

[mbt270436-bib-0041] Sabri, M. , K. El Handi , F. Valentini , et al. 2022. “Identification and Characterization of *Erwinia* Phage IT22: A New Bacteriophage‐Based Biocontrol Against *Erwinia amylovora* .” Viruses 14: 2455. 36366553 10.3390/v14112455 PMC9698647

[mbt270436-bib-0042] Schnabel, E. L. , and A. L. Jones . 2001. “Isolation and Characterization of Five *Erwinia amylovora* Bacteriophages and Assessment of Phage Resistance in Strains of *Erwinia amylovora* .” Applied and Environmental Microbiology 67: 59–64. 11133428 10.1128/AEM.67.1.59-64.2001 PMC92516

[mbt270436-bib-0043] Stenberg, J. A. 2017. “A Conceptual Framework for Integrated Pest Management.” Trends in Plant Science 22: 759–769. 28687452 10.1016/j.tplants.2017.06.010

[mbt270436-bib-0044] Timilsina, S. , N. Potnis , E. A. Newberry , et al. 2020. “ *Xanthomonas* Diversity, Virulence and Plant‐Pathogen Interactions.” Nature Reviews. Microbiology 18: 415–427. 32346148 10.1038/s41579-020-0361-8

[mbt270436-bib-0045] Tom, E. F. , I. J. Molineux , M. L. Paff , and J. J. Bull . 2018. “Experimental Evolution of UV Resistance in a Phage.” PeerJ 6: e5190. 30013847 10.7717/peerj.5190 PMC6042481

[mbt270436-bib-0046] Toussaint, B. , P. A. Munoz , and J. Pirnay . 2024. Overview and Outlook of Phage Therapy and Phage Biocontrol. Publications Office of the European Union.

[mbt270436-bib-0047] USFDA . 2025. Novel Drug Approvals for 2025|US Food and Drug Administration. U.S. Department of Health and Human Services. https://www.fda.gov/drugs/novel‐drug‐approvals‐fda/novel‐drug‐approvals‐2025.

[mbt270436-bib-0048] Vicente, J. G. , and E. B. Holub . 2013. “ *Xanthomonas campestris* pv. *campestris* (Cause of Black Rot of Crucifers) in the Genomic Era Is Still a Worldwide Threat to Brassica Crops.” Molecular Plant Pathology 14: 2–18. 23051837 10.1111/j.1364-3703.2012.00833.x PMC6638727

[mbt270436-bib-0049] Vique, G. , E. Mendoza‐Barberá , M. D. Ramos‐Barbero , et al. 2025. “Efficacy of *Erwinia amylovora* and *Xanthomonas campestris* pv *campestris* Phages to Control Fire Blight and Black Rot *in Vivo* .” Microbiol Spectr 13: e0028025. 40377312 10.1128/spectrum.00280-25 PMC12211020

