# Supplementary material for: Design of a Bacteriophage‐Based Product for Biocontrol of Fire Blight and Black Rot
Source: Microb Biotechnol. 2026 Sep 2;19(9):e70436. doi: 10.1111/1751-7915.70436 (PMC13535983; doi:10.1111/1751-7915.70436)
Supplement: Supplementary file 1 [file MBT2-19-e70436-s001.docx]

**Supplementary Table 1.** Inactivation parameters of phages ɸEF1, ɸEF2, and ɸXF1 at different pHs and temperatures. The best-fit model (segmental linear regression or one-phase decay) was selected based on the Akaike information criterion (AIC), r^2^, and root mean square error (RMSE). k and T_90_ are expressed in days^-1^.

| **Phage** | **pH/Tº** | **Best fitting model** | **k (days^-1^)**  **(95% CI)** | **Plateau**  **(95% CI)** | **r^2^** | **RMSE** | **Difference in AICc** | **T_90_ (days)**  **(95% CI)** |
| --- | --- | --- | --- | --- | --- | --- | --- | --- |
| ɸEF1 | pH 3 | Not calculated |  |  |  |  |  |  |
|  | pH7 | Linear regression | slope1 = -0.0266 (-0.0314 to -0.0219)  slope2 = -0.0022 (-0.0038 to -0.0006) |  | 0.7633 | 0.2031 | -109.0 | 37.5 (45.6 to 31.9)  460.2 (1726.2 to 265.5) |
|  | pH9 | Linear regression | slope1 = -0.0226 (-0.0273 to -0.0179)  slope2 = -0.0026 (-0.0042 to -0.0011) |  | 0.7334 | 0.2015 | -109.6 | 44.3 (55.9 to 36.6)  379.2 (946.9 to 237.1) |
|  | 4ºC | Linear regression | slope1 = -0.0266 (-0.0314 to -0.0219)  slope2 = -0.0022 (-0.0038 to -0.0006) |  | 0.7633 | 0.2031 | -109.0 | 37.5 (45.6 to 31.9)  460.2 (1726.2 to 265.5) |
|  | 20ºC | One-phase decay | 0.0609 (0.0470 to 0.0804) | -2.030 (-2.205 to -1.864) | 0.9089 | 0.2584 | 76.7 | 11.1 (8.4 to 14.4) |
|  | 37ºC | One-phase decay | 0.0214 (0.0168 to 0.0269) | -7.139 (-7.999 to -6.430) | 0.9484 | 0.4874 | 51.9 | 7.1 (5.6 to 9.0) |
| ɸEF2 | pH 3 | Not calculated |  |  |  |  |  |  |
|  | pH7 | Constant |  |  |  |  |  |  |
|  | pH9 | Constant |  |  |  |  |  |  |
|  | 4ºC | Constant |  |  |  |  |  |  |
|  | 20ºC | One-phase decay | 0.0414 (0.0295 to 0.0597) | -1.936 (-2.174 to -1.715) | 0.8748 | 0.2996 | 51.5 | 17.5 (12.2 to 24.6) |
|  | 37ºC | One-phase decay | 0.0138 (0.0102 to 0.0179) | -8.579 (-10.04 to -7.559) | 0.9524 | 0.5304 | 38.1 | 9.0 (6.9 to 12.1) |
| ɸXF1 | pH 3 | Not calculated |  |  |  |  |  |  |
|  | pH7 | One-phase decay | 0.1327 (0.0935 to 0.1975) | -1.645 (-1.769 to -1.527) | 0.8732 | 0.2323 | 87.7 | 7.1 |
|  | pH9 | One-phase decay | 0.0174 (0.0118 to 0.0245) | -3.490 (-4.165 to -3.024) | 0.8936 | 0.3807 | 31.7 | 19.4 |
|  | 4ºC | One-phase decay | 0.1249 (0.0880 to 0.1859) | -1.681 (-1.816 to -1.554) | 0.8823 | 0.2300 | 84.7 | 7.2 (4.9 to 10.3) |
|  | 20ºC | One-phase decay | 0.0181 (0.0136 to 0.0233) | -2.973 (-3.350 to -2.673) | 0.9320 | 0.2709 | 43.1 | 22.7 (17.6 to 30.2) |
|  | 37ºC | One-phase decay | 0.0562 (0.0349 to 0.0847) | -7.886(-10.09 to -6.643) | 0.9251 | 0.6827 | 23.3 | 2.4 (1.6 to 3.9) |

**Supplementary Table 2.** Inactivation parameters of phages ɸEF1, ɸEF2, and ɸXF1 under UV radiation and natural sunlight (SL) in liquid suspension or un vegetable surfaces (VS) with AA22 or CaCO_3_ at 12 and 24% as photoprotectants. The best-fit model (Linear regression or one-phase decay) was selected based on the Akaike information criterion (AIC), r^2^, and root mean square error (RMSE). k and T_90_ are expressed in hours^-1^.

| **Phage** | **Photoprotectant** | **Best fitting model** | **k (h^-1^)**  **(95% CI)** | **Plateau**  **(95% CI)** | ***r^2^*** | **RMSE** | **Difference in AICc** | **T_90_ (h)**  **(95% CI)** |
| --- | --- | --- | --- | --- | --- | --- | --- | --- |
| ɸEF1 | AA22 12%, UV | One-phase decay | 0.5290 (0.4704 to 0.5940) | -4.968 (-5.156 to -4.794) | 0.9916 | 0.1660 | 67.4 | 0.4 (0.4 to 0.5) |
|  | AA22 24%, UV | One-phase decay | 0.4079 (0.3062 to 0.5298) | -5.305 (-5.922 to -4.835) | 0.9616 | 0.3876 | 34.5 | 0.5 (0.4 to 0.7) |
|  | CaCO_3_ 12%, UV | Linear regression | -0.2413 (-0.2767 to-0.2058) |  | 0.7954 | 0.3201 | -2.7 | 4.1 (4.8 to 3.6) |
|  | CaCO_3_ 24%, UV | Linear regression | -0.1572 (-0.1876 to-0.1267) |  | 0.7211 | 0.2747 | -2.9 | 6.4 (7.9 to 5.3) |
|  | SL, VS | One-phase decay | 0.2831 (0.1668 to 0.4238) | -5.073 (-6.879 to -4.227) | 0.9689 | 0.2360 | 13.3 | 0.8 (0.5 to 1.3) |
|  | CaCO_3_ 24%, SL,VS | One-phase decay | 0.6391 (0.2528 to NC) | -2.865 (-4.073 to -2.283) | 0.7657 | 0.4861 | 8.4 | 0.7 (NC to 1.7) |
| ɸEF2 | AA22 12% | One-phase decay | 0.6176 (0.5476 to 0.6937) | -4.393 (-4.535 to -4.260) | 0.9930 | 0.1355 | 71.2 | 0.4 (0.4 to 0.5) |
|  | AA22 24% | One-phase decay | 0.7057 (0.4903 to 0.9908) | -3.365 (-3.352 to -2.819) | 0.9390 | 0.2889 | 35.9 | 0.6 (0.4 to 0.8) |
|  | CaCO_3_12% | Linear regression | -0.2449 (-0.2618 to-0.2281) |  | 0.9571 | 0.1521 | -6.2 | 4.1 (4. 4 to 0.8) |
|  | CaCO_3_24% | Linear regression | -0.1618 (-0.1955 to-0.1280) |  | 0.5536 | 0.3052 | -0.7 | 6.2 (7.8 to -5.0) |
|  | SL, VS | Linear regression | -0.8142 (-0.9423 to -0.6862) |  | 0.7752 | 0.7338 | -0.2 | 1.2 (1.5 to-1.1) |
|  | CaCO_3_24%, SL, VS | One-phase decay | 0.7788 (0.2798 to ???) | -2.770 (-3.891 to -2.208) | 0.7364 | 0.5032 | 8.2 | 1.8 (2.5 to-1.4) |
| ɸXF1 | AA22 12% | One-phase decay | 0.1905 (0.1356 to 0.2505) | -4.654 (-5.708 to -4.030) | 0.9824 | 0.1785 | 26.1 | 1.3 (1.0 to 1.8) |
|  | AA22 24% | One-phase decay | 0.3588 (0.2769 to 0.4539) | -4.116 (-4.576 to -3.773) | 0.9740 | 0.2313 | 36.9 | 0.8 (0.6 to 1.0) |
|  | CaCO_3_12% | Linear regression | -0.2961 (-0.3134 to -0.2788) |  | 0.9724 | 0.1565 | -2.9 | 3.4 (3.6 to 3.2) |
|  | CaCO_3_24% | Linear regression | -0.1547 (-0.1714 to -0.1379) |  | 0.8986 | 0.1509 | -2.9 | 6.5 (7.2 to 5.8) |
|  | SL, VS | One-phase decay | 1.326 (1.077 to 1.641) | -4.649 (-4.978 to -4.347) | 0.9661 | 0.2592 | 42.9 | 0.2 (0.1 to 0.2) |
|  | CaCO_3_24%, SL, VS | One-phase decay | 0.4028 (0.2648 to 0.5721) | -5.027 (-6.150 to -4.349) | 0.9402 | 0.3539 | 21.7 | 0.5 (0.34 to 0.8) |

UV: ultraviolet radiation SL:natural sunlight, VS: vegetal surface, NC: not calculated
